# Automated Vulnerability Discovery and Exploitation in the Internet of Things †

**DOI:** 10.3390/s19153362

**Published:** 2019-07-31

**Authors:** Zhongru Wang, Yuntao Zhang, Zhihong Tian, Qiang Ruan, Tong Liu, Haichen Wang, Zhehui Liu, Jiayi Lin, Binxing Fang, Wei Shi

**Affiliations:** 1 Key Laboratory of Trustworthy Distributed Computing and Service (Beijing University of Posts and Telecommunications), Ministry of Education, Beijing 100876, China; 2 Cyberspace Institute of Advanced Technology, Guangzhou University, Guangzhou 510006, China; 3 Beijing DigApis Technology Co., Ltd, Beijing 100081, China; 4 School of Information Technology, Carleton University, Ottawa, ON K1S 5B6, Canada

**Keywords:** security, vulnerability discovery, vulnerability exploitation

## Abstract

Recently, automated software vulnerability detection and exploitation in *Internet of Things* (IoT) has attracted more and more attention, due to IoT’s fast adoption and high social impact. However, the task is challenging and the solutions are non-trivial: the existing methods have limited effectiveness at discovering vulnerabilities capable of compromising IoT systems. To address this, we propose an Automated Vulnerability Discovery and Exploitation framework with a Scheduling strategy, *AutoDES* that aims to improve the efficiency and effectiveness of vulnerability discovery and exploitation. In the vulnerability discovery stage, we use our *Anti-Driller* technique to mitigate the “path explosion” problem. This approach first generates a specific input proceeding from symbolic execution based on a *Control Flow Graph* (CFG). It then leverages a mutation-based fuzzer to find vulnerabilities while avoiding invalid mutations. In the vulnerability exploitation stage, we analyze the characteristics of vulnerabilities and then propose to generate exploits, via the use of several proposed attack techniques that can produce a shell based on the detected vulnerabilities. We also propose a genetic algorithm (GA)-based scheduling strategy (AutoS) that helps with assigning the computing resources dynamically and efficiently. The extensive experimental results on the RHG 2018 challenge dataset and the BCTF-RHG 2019 challenge dataset clearly demonstrate the effectiveness and efficiency of the proposed framework.

## 1. Introduction

With the rapid development of the *Internet of Things* (IoT) [1,2,3], more and more emerging software programs and applications are being designed and developed. It opens opportunities for IoT devices and software programs to share and communicate information on the Internet [4]. As a result, proper protection on such rapidly designed and developed software programs become a pressing concern. The Internet of Things [5] suffers from a variety of attacks [6], thus the vulnerability discovery and exploitation become the key technologies. More specifically, the number of newly detected software vulnerabilities in *Internet of Things* increased drastically and dramatically. Recently, automated vulnerability discovery and exploitation methods [7] have attempted to solve the security problems about software vulnerabilities automatically. However, the existing methods cannot be applied or extended for software vulnerabilities in the IoT, due to the following reasons.

First, the conventional vulnerability discovery and exploitation methods mainly focus on the system architectures of the traditional PC industry, such as x86 machine architecture. Due to the high occupancy of these systems, developers implement a large number of software programs on them, where the corresponding security awareness attracts more attention than that of the IoT. In addition, in the IoT, diversified hardware platforms and customized operating systems make it difficult for programmers to protect the IoT security.

Second, with the explosive increase of software complexity, it is rigorous for software developers to take care of every aspect of secure programming. This leads to many IoT devices having vulnerabilities that are exposed to attackers. Consequently, attackers may plant backdoors in a vulnerable device by attackers to control the device. However, the number of software programs on the IoT devices increases exponentially and it is costly to deal with these vulnerabilities manually one by one. Thus, an automated framework is required.

Last but not least, challenges remain since the existing vulnerability discovery and exploitation methods, such as fuzzing [8], taint analysis [9] and concolic execution [10], are not scalable, which may lead to the failure on detecting and exploiting vulnerabilities in large scale IoT systems. Thus, improving the effectiveness of these methods is necessary.

To tackle the above challenges, researchers turn to seek attacks using automated vulnerability discovery and exploitation approaches for the IoT security. For example, in 2006, DARPA held the Cyber Grand Challenge (CGC), where each team used its cyber reasoning system (CRS) to automatically identify software flaws. Later on, following CGC, the Robo Hacking Game (RHG) took place in China in 2018. Many automated vulnerability analysis systems, such as Driller [11], are also designed to automatically discover software vulnerabilities. However, how to improve the effectiveness and *the efficiency* of vulnerability discovery and exploitation are still challenging tasks. In the rest of this section, we further review major challenges related to existing methods and attacks, followed by a summary of our main contributions.

### 1.1. Challenge 1: Improvement on Vulnerability Discovery

The existing vulnerability discovery methods can be divided into three categories that are static analysis, fuzzing and concolic execution. Static analysis can provide provable guarantees without executing software programs. For example, GUEB [12] detects use after free on binary code statically. However, this method cannot provide valuable runtime information as well as specific inputs that can trigger detected vulnerabilities. Fuzzing methods require little knowledge about the target software programs. For example, AFL [13] automatically discovers interesting test cases that trigger new internal states in the targeted binary without any knowledge about that binary. Such methods need “test cases” as input when verifying a software program, and they are often limited by the coverage of branches in target software programs due to the lack of actual input that should be in an expected format. Dynamic symbolic execution methods generate inputs to explore the state space of the target software based on the software interpretation and symbolic constraint techniques. It can trigger a large number of execution paths in a software program. For example, EXE [14] tracks the constraints on each symbolic memory location instead of running code on manually or randomly constructed input while KLEE [15] is a variant of EXE, that employs a variety of constraint solving optimizations and uses search heuristics to get high code coverage. Unfortunately, dynamic symbolic execution suffers from serious problems, such as “path explosion” that limits its scalability. Consequently, to address the above drawbacks, Driller [11], built on both fuzzing and symbolic execution techniques, was proposed. However, as Driller uses a mutation-based fuzzing method to explore a software program, it may find vulnerabilities that are located on unconcerned functions, such as library functions. Furthermore, the fuzzer may not mutate qualified inputs for the intended objective functions.

To take full advantage of different discovery methods, in the vulnerability discovery stage of our solution, we propose alleviating the “path explosion” problem by analyzing the control flow graph of a target program, and then providing good input test cases for fuzzers to skip unconcerned functions.

### 1.2. Challenge 2: Improvement on Vulnerability Exploitation

There exist methods with the goal of exploiting detected vulnerabilities, such as AEG [16] and Rex [7]. However, these methods are limited by the following two issues:

1. these methods can collect run-time information of the specified software when providing the source codes, which may not be available in practice. For example, AEG necessarily requires a manual preprocessing, which compiles the source codes to a binary form.
2. since our targets are real-life applications, the existing methods fall short of effectiveness. For example, Rex cannot be directly applied onto the real-life applications, as it is designed for a specific CGC challenge.

In the vulnerability exploitation stage of our solution, based on the detected vulnerabilities, we propose producing a shell with multiple attack techniques, including *Injecting a ShellCode* [17], *Return Oriented Programing* [18] and *Jmp Esp* [19]. We provide more details in Section 4.2.

The above challenges also exist in the IoT environments. For example, Xiao et al. [20] propose that few effective methods are established in terms of vulnerability discovery and exploitation, especially for vulnerabilities in software or firmware of Industry Internet of Things. IOTFUZZER [21] leverages a taint-based fuzzing approach. RPFuzzer [22] aims to detect vulnerabilities in routers, which monitors routers by sending normal packets, keeping an eye on CPU utilization and checking system logs. DrE [23] uses a symbolic execution method targeting the sensor input channel of an embedded system and generates traces of sensor readings that will drive an MSP430-based embedded system to a chosen point in its code. However, how to improve the effectiveness and the efficiency remains a problem.

### 1.3. Challenge 3: Efficient Scheduling Solution

As we aim to detect and exploit vulnerabilities automatically, an efficient scheduling solution that assigns the computing resources dynamically and efficiently is required. Unfortunately, we cannot simply apply or extend any existing algorithm for the scheduling due to the following reasons: first, the problem of finding an optimal scheduling solution is *NP-Complete*. It is computationally intractable to find a global optimal solution. Second, an IoT network consists of many low-power, low-cost, and small-size network nodes [1,24,25,26,27]. The scheduling cost must be reduced as much as possible. Third, the conventional scheduling methods, such as [28], often fall short in efficiency.

In Table 1, we summarize the similarity and difference by comparing the existing method and our proposal.

To fill this gap, we propose a framework **AutoDES**, to integrate automatic vulnerability discovery (**AutoD** for short) and automatic vulnerability exploitation (**AutoE** for short) methods with an efficient scheduling strategy (**AutoS** for short). Please note that we focus on the binary files only and our framework can also be extended to the binaries in the IoT environment (see the case study in Section 5.3) The main contributions of this paper are as follows:

First, in order to improve the effectiveness of vulnerability discovery, in AutoD stage, we propose a novel method, *Anti-Driller*. Unlike Driller, it first uses a concolic execution engine to find a specific path, then generates a specific test case by avoiding other program states. It then leverages a mutation-based fuzzer to explore the software by using its test case as input and mutates it to determine whether there exists any vulnerability. This method achieves good performance and reduces the time cost.

Second, to improve the effectiveness of vulnerability exploitation, in AutoE stage, we propose three attack methods, *IPOV fuzzer*, *AutoROP*, and *AutoJS*. Specifically, IPOV fuzzer overwrites the correct address of the *return address* with a shellcode, AutoROP leverages the *Return Oriented Programming* attack technique and AutoJS leverages the *Injecting a ShellCode* and *Jmp Esp* techniques, respectively. With the help of an increasing number of detected vulnerabilities, these three methods enable successful exploitations.

Third, to allocate computing resources dynamically and efficiently, we propose an efficient genetic algorithm (GA)-based scheduling solution: *AutoS*, which produces a scheduling solution by optimizing a specific fitness function. Comparing to the existing scheduling methods, AutoS improves on the efficiency of vulnerability detection.

Finally, we report an extensive experimental study running on the RHG 2018 challenge dataset as well as the BCTF 2019 challenge dataset. The results clearly show that the effectiveness and efficiency of the proposed framework.

The remainder of this paper is organized as follows: we review the related work in Section 2. The overview of AutoDES is introduced in Section 3. We present a detailed implementation in Section 4. We report the evaluation results in Section 5. Finally, we conclude the paper in Section 6.

## 2. Related Work

In this section, we review the related work. There are three categories of studies related to our work: feedback and concolic execution based vulnerability discovery, automated exploit generation, resource scheduling algorithms and IoT security.

### 2.1. Feedback and Concolic Execution Based Vulnerability Discovery

Feedback based vulnerability discovery inputs possible magic values with their corresponding positions and makes heuristics based on the feedback from the target software programs [39]. Although it is efficient, it may fall short in finding new paths that should be executed. In order to efficiently increase the code coverage, AFL [13] is proposed to mutate the test cases. Consequently, AFLFast [29] and AFLGo [30] are proposed to improve the performance. AFLFast suggests a new power schedule that spends more energy on low-frequency paths and less energy on high-frequency paths. AFLGo generates inputs with the object of reaching a given set of target software locations efficiently. Steelix [31] proposes to locate the magic bytes in the test input and then mutates the specific input to match the magic bytes efficiently.

Dynamic symbolic execution, which was first introduced in EXE [14], uses symbolic variables to model the user input and then uses constraint solvers to create inputs for driving software programs down specific paths. Consequently, KLEE [15] refines it. Due to the high coverage of executed paths, SAGE [33], DART [34], CUTE [35], Smart-Fuzz [36] and Driller [11] that leverage concolic execution techniques are proposed.

Different from other approaches, Driller uses selective concolic execution. That is, it first uses a fuzzer to explore the software programs, and further uses a concolic execution engine to guide the fuzzer when the fuzzer cannot find a new path. However, the Driller may suffer in scalability to cover paths protected by checks. To solve the above challenges, T-Fuzz [32] is proposed, which differs from Driller in improving the bug finding ability of a fuzzer by disabling input checks in the software.

However, the feedback based methods are limited by the coverage of branches in the specific software while the concolic execution-based methods suffer from “path explosion”. In this paper, we propose improving the effectiveness of the vulnerability discovery by generating a specific input with a concolic execution engine first and then using this input as the initial compartment to explore the possible inputs and find the potential vulnerabilities.

### 2.2. Automated Exploit Generation

Automatic exploit generation is an important technique that can prevent an attacker from executing arbitrary code on a hacked computer. Brumley et al. [37] propose a patch-based exploit generation method, named by **APEG**. However, they only consider the exploit as an input violating a new safety check introduced by a patch. Avgerinos et al. [16] extend the notion of the exploit and propose a control flow hijacking exploit generation method, named by **AEG**. AEG first locates the vulnerability, and then collects run-time information of a software program. It further generates and verifies the exploits automatically. Shoshitaishvili et al. [7] propose **Rex** and use it for CGC challenges.

However, as AEG needs a necessary manual preprocessing and Rex is designed for CGC challenges, they cannot be directly applied on the real-life applications that we mainly focus on.

### 2.3. Resource Scheduling Algorithms

Scheduling algorithms, such as Minimum-Minimum completion time algorithm [40], Minimum Completion Time algorithm [41] and Round-Robin Scheduling algorithm [38], are widely used to allocate the computing resources. These algorithms are easy to be implemented, but fall short in efficiency. To tackle the above issue, many heuristic methods, such as Genetic Algorithm-based methods [42], Artificial Bee Colony Algorithm-based methods [43] and Simulated Annealing Algorithm-based methods [44], are proposed. These methods commonly have strong adaptability and can always achieve good performances.

Compared with the scheduling method of Mechanical Phish [7], our resource scheduling can allocate the computing resources dynamically and optimally, and thus our method can improve the efficiency of vulnerability detection. After the vulnerability detection, the cyber range [45] can be used to reappear the vulnerability and blockchain [46] and Evidence Reasoning Network [2] can be used to manage the vulnerabilities.

### 2.4. IoT Security

With the increasing of IoT devices and software programs, there are already efforts on detecting security vulnerabilities in IoT. As there are various methods, here we mainly focus on the related studies that discover and exploit vulnerabilities on binaries, such as firmware images.

Costin et al. [47] propose to discover many vulnerabilities by application level emulation and static analysis manually. Chen et al. [48] extend Costin’s work by emulating the whole file systems of Linux-based firmware images with Qemu. Zaddach et al. [49] propose Avatar, a framework that enables the complex dynamic analysis of embedded devices by orchestrating the execution of an emulator together with the real hardware.

In contrast to the above studies, our proposal focuses on *an automated manner* that can first discover vulnerabilities and then exploit them.

Next, we will introduce the vulnerability discovery and exploitation techniques in detail.

## 3. Overview of the Framework

In this section, we give an overview of the proposed framework AutoDES. It follows the architecture of Mechanical Phish, which is first used in the CGC Final Event. Next, we will first show the overall procedure of AutoDES and then illustrate the difference between Mechanical Phish and our proposed framework.

First, we briefly review the components of Mechanical Phish. Please note that we only review those contained in both Mechanical Phish and AutoDES. *Ambassador* interacts with external components, which collects the testing software programs and submits the feedback. *Farnsworth* provides data storage services for all corresponding data, such as binary software programs, proof of vulnerabilities and crashes. *Meister* schedules different tasks and determines which tasks should be executed based on the priority information in terms of memory and CPU. *AFL* and *Driller* are used to discover vulnerabilities while *POV fuzzer* and *Rex* generate exploits.

**Figure 1:**
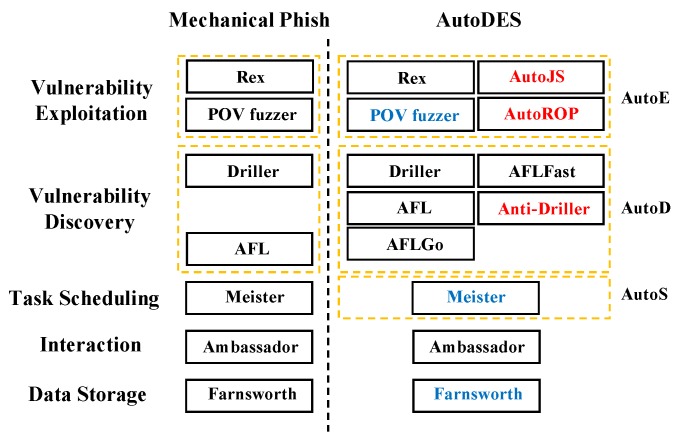
The difference between AutoDES and Mechanical Phish, where the red text represents the newly added modules and the blue text represents the improved ones. We omit the unused and unchanged parts of Mechanical Phish.

The overall procedures of AutoDES and Mechanical Phish are similar: they both collect testing software programs, discover vulnerabilities, generate exploits then submit feedbacks. However, as Mechanical Phish is designed for CGC, it falls short in discovering and exploring the vulnerabilities of the real-life software programs. As shown in Figure 1, the difference between AutoDES and Mechanical Phish can be concluded as follows:

1. **Difference on Data Storage:** Mechanical Phish includes more functions than AutoDES, such as patching. It consumes more space to store data. To maintain a much lighter weight in terms of space cost by avoiding storage for unnecessary data, AutoDES decreases about 70.6% unnecessary data storage by comparing with Mechanical Phish. This makes its database structure more concise than that of Mechanical Phish.
2. **Difference on resource scheduling:** Mechanical Phish uses *Meister* to determine how to run different jobs based on the priority of each job. However, it is not efficient in vulnerability detection. Different from the resource scheduling scheme used in Meister, AutoDES uses a GA-based scheduling method that helps decrease the vulnerability detection time cost.
3. **Difference on vulnerability discovery:** Mechanical Phish uses AFL and Driller for vulnerability discovery. Instead, AutoDES involves two variants of AFL, i.e., AFLGo and AFLFast. Moreover, AutoDES adopts a new method Anti-Driller that first uses a concolic execution engine to find a specific path and then generate a specific test case by avoiding other program states. Then, it leverages a mutation-based fuzzer to explore the software by using this test case as the initial input and mutating it to determine whether there exists any vulnerability. Comparing to Mechanical Phish, AutoDES improves the effectiveness of vulnerability discovery.
4. **Difference on vulnerability exploitation:** Mechanical Phish employs two exploitation modules: POV fuzzer and Rex. Given a crashing input, POV fuzzer tracks the relationship between input bytes and registers at the crash point, while Rex symbolically executes the input and tracks formulas for all registers and memory values. However, these two modules do not work very well in practice. To improve the effectiveness of vulnerability exploitation, AutoDES further improves the POV fuzzer (the improved POV fuzzer is referred to as IPOV fuzzer here after), such that it also works well on simpler/smaller software programs. Furthermore, we propose AutoROP and AutoJS to generate exploits and finally produce a shell.

The overall procedure of AutoDES is listed in Algorithm 1 and summarized as follows: given a remote binary software program, Ambassador retrieves it as *p* such that it can be analyzed locally (Line 1). After that, Meister schedules *p* and assigns computing resources for it (Line 2). For a simple software program, the IPOV fuzzer can generate a successful exploit and produce a shell faster than other methods. We first invoke an IPOV fuzzer and determine whether it can produce a shell successfully (Lines 3–4). Unfortunately, the IPOV fuzzer may not work well and we further leverage AutoD and AutoE to discover and exploit vulnerabilities, respectively (Lines 5–14). In the stage of AutoD, we use multiple methods Driller, AFL, AFLGo, AFLFast and Anti-Driller to detect vulnerabilities and generate crashing inputs (Line 6). Then, in the stage of AutoE, we use multiple methods Rex, AutoJS and AutoROP to check each crashing input iteratively (Lines 7–13). Once we produce a shell, this process terminates immediately to avoid redundant checks for other crashing inputs (Lines 10–11). Note that not every crashing input can lead to a successful exploit, and thus we may not necessarily produce a shell at the end of each process either. Finally, we output a shell or ∅.

Next, we describe the detailed techniques for vulnerability discovery and exploitation.    

**Algorithm 1:** The overview of AutoDES **Input**: A remote binary software **Output**: A shell or ∅1:p←Ambassador(software);2:Assign computing resources for *p* with Meister;3:exploit=IPOVfuzzer(p);4:shell=get_a_shell(exploit);5:**if**shell==NULL**then**6: crashinginputs←AutoD(p);7: **for** Each crash∈crashinginputs **do**8:  exploit=AutoE(crash);9:  shell=get_a_shell(exploit);10:  **if** shell≠NULL **then**
11:   break;12:  **end if**13: **end for**
14:**end if**15:**return**Ashell or ∅;

## 4. Implementation

In this section, we introduce the implementation detail of AutoDES in different stages.

### 4.1. AutoD: Automated Vulnerability Discovery

Although Driller has achieved good performance, it still falls short of efficiency. For example, as shown in Listing 1, to trigger the bug located on Line 12, Driller must bypass the sanity check on Line 5. However, it is difficult for Driller to bypass this check, as it uses fuzzing, which needs a good input test case.

**Figure 2:**
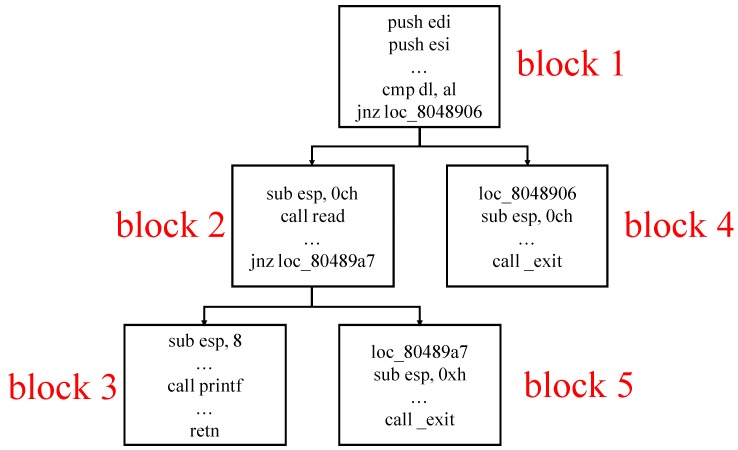
The control flow graph for Listing 1.

To overcome the above challenge, we propose Anti-Driller, which differs from Driller: it first uses a concolic execution engine to generate a specific test case as input and then leverages a mutation-based fuzzer to mutate this test case to find vulnerabilities. Specifically, we construct a CFG, where the nodes represent basic blocks of instructions and the directed edges represent control flow transfers between two blocks, to alleviate the “path explosion” problem. By traversing the CFG in a depth-first order, we can obtain a specific path in each traversal. For example, Figure 2 shows a CFG for Listing 1. By traversing it in a depth-first order, it first finds block 1, block 2 and block 3 sequentially ignoring blocks 4 and 5. This enables reduction on the space complexity.

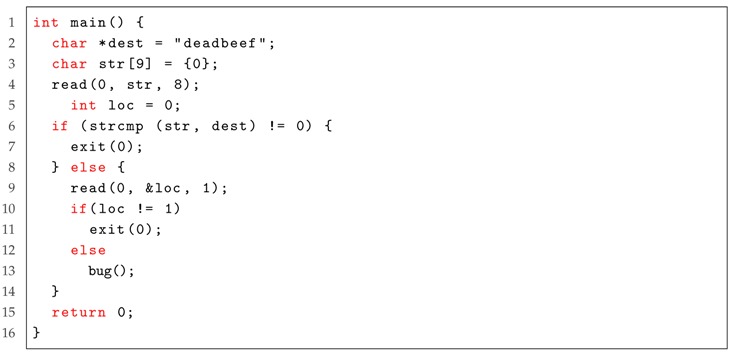
Listing 1: An example software for Anti-Driller.

The process of Anti-Driller is outlined in Algorithm 2. We first initialize a stack *S* and Φ (Line 1). Then, we construct a CFG *G* for the software *p* (Line 2). As *p* has only one entry, we use $n_{0}$ to represent the root node of *G* and push it to stack *S* (Line 3). If the stack is not empty, we pop a node $n_{i}$ from stack *S* (Line 4–5). For each unvisited adjacent node $n_{j}$ of $n_{i}$, we check if $n_{j}$ has any child (Line 7). If $n_{j}$ has no children, we obtain an execution path $path=\langle n_{0},\ldots ,n_{i},n_{j}\rangle$ (Line 8). It then generates a specific test case ans (Line 9). Then, the fuzzer uses ans as the initial input to generate and mutate crashing inputs and each crashing input *s* is added into Φ (Line 10–12). Otherwise, if $n_{j}$ has at least a child, it will be pushed into stack *S* (Line 14). Finally, the crashing inputs Φ is returned as the final output.

In summary, Anti-Driller improves Driller by bypassing the sanity check which is difficult for Driller. However, how to generate exploits and obtain the shell automatically remains a problem. Next, we will introduce the details of the automated vulnerability exploitation and explain how to use the crashing inputs of AutoD.    

**Algorithm 2:** Anti-Driller **Input**: A software *p*
 **Output**: Crashing inputs Φ1:Initial a stack S←∅, Φ←∅;2:Construct a CFG *G* for *p*;3:$S\leftarrow n_{0}$;4:**while**S≠∅**do**5:$\;n_{i}=S.destack\left(\right)$;6: **for** Each unvisited adjacent node $n_{j}$ of $n_{i}$ **do**7:  **if** $n_{j}$ has no children **then**8:$\;path=\langle n_{0},\ldots ,n_{i},n_{j}\rangle$;9:   ans=concolic_execution_solver(p,path);10:   **for** Each crashing input *s* produced by fuzzer(ans) **do**11:    Φ←Φ∪s;12:   **end for**13:  **else**14:$\;S.instack(n_{j})$;15:  **end if**16: **end for**17:**end while**18:**return**Φ;

### 4.2. AutoE: Automated Vulnerability Exploitation

In this section, we propose three efficient vulnerability exploitation attacks: IPOV fuzzer, AutoJS and AutoROP.

#### 4.2.1. IPOV Fuzzer

The improved POV (IPOV) fuzzer is an elegant and efficient attack on simple software programs. The key idea of improved IPOV fuzzer is that, in a stack-overflow return-to-stack attack, if we can obtain the correct address of the *return address*, we may overwrite it with a specific shellcode and then achieve a successful exploit.

The exploitation using IPOV fuzzer is outlined in Algorithm 3. Given a specific software and a crashing input, our goal is to produce a shell. The crashing input could be user-specific, such as a long string where every four zero-based bytes of this string are different. This enables us to receive the return address. We can also use a crashing input generated by any method in the AutoD stage. A specific crashing input will be used as the input for the attack (Line 1). When the software is crashed, the system will produce a core dump file automatically and the run-time information of registers and memory can be obtained from this file. The correct offset is obtained using a long common string algorithm matching the crashing input (Lines 2–3). Combining the offset and the shellcode, an exploit is built (Line 4) and a shell is produced as the final output (Lines 5–6).    

**Algorithm 3:** POV fuzzer **Input**: A software and a crashing input
 **Output**: A shell or ∅1:Use the crashing input as the input for the software;2:c=read(core_dump_file);3:offset=long_common_str(c);4:exp_str←offset+shellcode;5:shell=get_a_shell(exp_str);6:**return**Ashell or ∅;

Now we use an example to further explain the process of IPOV fuzzer. As shown in Listing 2, a vulnerability locates in function *read()*. We input a long string, i.e., “AAA%AAsAABAA$A AnAACAAAA(AADAA;AA)AAEAAaAA0AAFAAbAA1AAGAAcAA2AAHAAdAA3AAIAAeAA4A AJAAfAA5AAKAAgAA6AALAAhAA7AAMAAiAA8AANAAjAA9AAOAAkAAPAAlAAQAAmAA RAAoAASAApAATAAqAAUAArAAVAAtAAWAAuAAXAAvAAYAAwAAZAAxAAyA”, and this software is crashed. By reading the “core.dump” file, we find that the the content of register eip is “rAAV”. We then calculate the offset between the starting address of the input string and the return address by using a longest common substring algorithm, such as [50]. At the end of the offset, we attach a shellcode and finish creating an exploit.

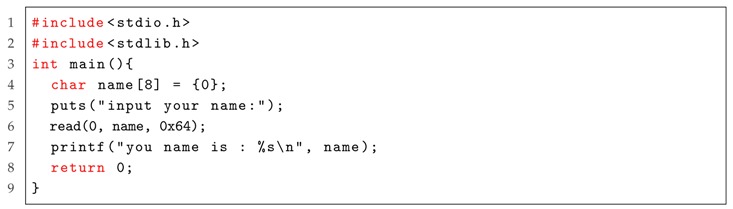
Listing 2: An example software, where the vulnerability locates in function *read()*.

#### 4.2.2. AutoJS

In this section, we introduce how AutoJS generates an exploit for bypassing stack randomization. The key idea here is that AutoJS overwrites the return address of a function and makes the program counter point back to the injected input, e.g., a shellcode.

The process of AutoJS is outlined in Algorithm 4. Given a specific software *p* and a crashing input *s*, we first obtain the offset between the return address and the start address (Line 1). Then, the *jmp_esp_address* of the specific assembly code “jmp esp” is searched in the disassemble program of *p* (Line 2). This address will be used as a springboard for filling the shellcode. Upon successfully obtaining the address, an exploit construction begins (Lines 4–7). The exploit consists of an overwritten string (i.e., the crashing input, Lines 4–6), the *jmp_esp_address* (Line 7) and the shellcode (Line 7). Finally, a shell (Line 8) is produced as the output (Line 10). Using Listing 2 as an example: given a crashing input, whose length equals to 20, we obtain the *jmp_esp_address*: (0x${080ac99c)}_{16}$. Then, we attach a specific shellcode to produce a shell.    

**Algorithm 4:** AutoJS **Input**: A software *p*, a crashing input *s* and a shellcode
 **Output**: A shell or ∅1:offset=get_offset(p,s);2:jmp_esp_address=search(“jmpesp”,p);3:**if**jmp_esp_address!=NULL**then**4: **for** i=1 to offset **do**5:  exploit[i++]=s[i];6: **end for**7: exploit←exploit+jmp_esp_address+shellcode;8: shell=get_a_shell(exploit);9:**end if**10:**return**Ashell or ∅;

#### 4.2.3. AutoROP

As executable-space protection marks memory regions as non-executable, the shellcode cannot execute, therefore, AutoJS does not work. To tackle this problem, a *Return-Oriented Programming* technique is proposed [51]. The key idea is to create short instruction streams that exist in the software. For example, as we would like to use the function “execve(‘/bin/sh’, NULL, NULL)” to produce a shell, we should first put the string “/bin/sh” on the stack, and then set *EAX*, *EBX*, *ECX* and *EDX* in the registers to “0xb”, the address of string “/bin/sh”, 0 and 0, respectively. With these short instruction streams, we no longer need to inject and execute a shellcode. Next, we explain how to use this technique to generate an exploit automatically.

The process of AutoROP is outlined in Algorithm 5. Given a specific software *p* and a crashing input *s*, we first obtain the offset between the return address and the start address (Line 1). Then, we find the ROP gadgets contained in *p* (Line 2). Upon successfully obtaining the address, the exploit (Lines 4–7) construction begins. The exploit consists of an overwritten string (i.e., the crashing input, Lines 4–6) and the ROP gadgets (Line 7). Finally, a shell (Line 8) is produced as the output (Line 10).    

**Algorithm 5:** AutoROP **Input**: A software *p* and a crashing input *s*
 **Output**: A shell or ∅1:offset=get_offset(p,s);2:rop_gadgets=find_rop(p);3:**if**rop_gadgets!=NULL**then**4: **for** i=1 to offset **do**5:  exploit[i++]=s[i];6: **end for**7: exploit←exploit+rop_gadgets;8: shell=get_a_shell(exploit);9:**end if**10:**return**Ashell or ∅;

Now, we explain the process of AutoROP on exploiting the vulnerability using Listing 2 as an example. The exploit is shown in Listing 3. With the crashing input in Line 1 of Listing 3, an execution command, i.e., “/bin/sh”, is put on the stack. Then, the addresses of assembly instructions “pop EAX”, “pop EBX”, “pop ECX”, “pop EDX” and their crashing input are searched, respectively. These instructions will be executed sequentially. Finally, the syscall “execve(‘/bin/sh’, NULL, NULL)” is executed in order to produce a shell.

In summary, AutoE produces three types of exploits, which are the most popular classic control hijack attack techniques. In addition, AutoE allows bypassing **Stack No-eXecute Protection** and stack randomization. As our framework may use multiple methods to discover and exploit vulnerabilities simultaneously, it requires assigning the limited computing resources for multiple methods. Next, we will introduce the scheduling strategy based on the priority of each job, where a job is an instance of a specific discovery or exploitation method.

### 4.3. AutoS: A GA-Based Scheduling Strategy

In AutoDES, the process for a specific method of vulnerability discovery and exploitation is considered as a *task*. As the total computing resources for AutoDES is limited, it must assign some resources, such as the cores of CPUs and the main memory, for a specific binary. In this section, we propose AutoS, an efficient scheduling solution that can assign such resources dynamically.

Inspired by biological evolution, genetic algorithms are widely used in solving *NP-Complete* problems. In AutoS, a scheduling sequence is treated as a chromosome of an individual, each of which has its own fitness value. As the genetic algorithms run in an iterative manner, chromosomes in each generation include the survivors of the previous generation as well as the new superior chromosomes that are newly created after a selection, crossing and mutation cycle. Chromosomes that have greater fitness values than others are selected as a result. This process iterates until a given set of termination conditions are satisfied.

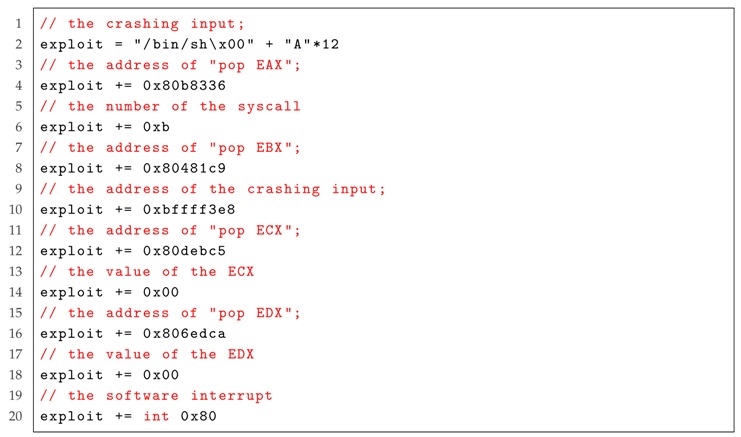
Listing 3: The exploit of AutoROP for the program in Listing 2.

We use an example to further explain the above-mentioned strategy. Given five tasks and three computing nodes, the length of the chromosome will be 5 and the value of each gene is randomly generated from 0 to 2. For example, {2, 0, 1, 1, 2} is a randomly generated chromosome, and this chromosome indicates that the first task will run on the second computing node. The task scheduling strategy that we adopt for AutoDES is as follows: given the task set $T=\{t_{1},t_{2},\ldots ,t_{n}\}$ and the computing nodes $VM=\{vm_{1},vm_{2},\ldots ,vm_{m}\}$, the task scheduling problem is to assign *n* tasks to *m* computing nodes for execution (m<n). The relationship between tasks and computing nodes can be described as a metric, referred to as ETC. It is shown in Equation (1), where $ETC_{i,j}$ represents the expected execution time of the task $t_{i}$ on a computing node $vm_{j}$:

(1) $$\left(\begin{array}{ccc} ETC_{11} & ETC_{12} & \ldots \;ETC_{1m}\\ ETC_{21} & ETC_{22} & \ldots \;ETC_{2m}\\ \ldots & \ldots & \ldots \\ ETC_{n1} & ETC_{n2} & \ldots \;ETC_{nm} \end{array}\right).$$

For example, given five tasks with their corresponding task cost {3000,400,1200,8000,20,000} and three computing nodes with their corresponding computing resources {400,1000,2500}, the ETC matrix is shown in Label (2):

(2) $$\left(\begin{array}{ccc} 7.5 & 3 & 1.2\\ 1 & 0.4 & 0.16\\ 3 & 1.2 & 0.48\\ 20 & 8 & 3.2\\ 50 & 20 & 8 \end{array}\right).$$

Based on the ETC matrix, we define the fitness function fit(k), which is shown in Equation (3). If a chromosome has a greater fitness value than others, it gets a greater probability to be selected:

(3) $$fit\left(k\right)=\frac{1}{\sum_{i=1}^{n}ETC_{i,j}}.$$

The process of AutoS is outlined in Algorithm 6. We initial set, $set_{1}$, $set_{2}$ and result as empty sets (Line 1). Then, α chromosomes are selected randomly (Line 2) and the ETC matrix is computed according to Equation (1) (Line 3). The fitness values for each solution according to Equation (2) and the number of selected solutions are obtained (Lines 5–6). select_num chromosomes from set are selected in ascending order of their fitness values then put into set_1 (Lines 7–8). *Y* will be selected from set_1 with the highest fitness value and put into result (Lines 8–9). The processes of crossover operator and mutation operator are shown in Lines 10–15 and Lines 16–20, respectively. For the crossover operator, in order to select chromosomes effectively, we use a single point intersection. That is, we select two individuals *x* and *y* from set randomly (Line 11), cross the randomly selected point of *x* and *y* (Line 12), and then generate two new chromosomes n_x and n_y (Line 13). The newly generated chromosomes are added into set_2 (Line 14). For the mutation operator, we first select the mutation point mutate_point randomly (Line 17). Then, for each chromosome in set_2, it is then determined to mutate with a probability γ (Line 18). set_2 is updated with the newly generated chromosomes tmp (Line 19). After that, set is updated as the union of set_1 and set_2. This process iterates for δ rounds. Finally, the solution *X* is selected and added to result with the highest fitness value.

**Figure 3:**
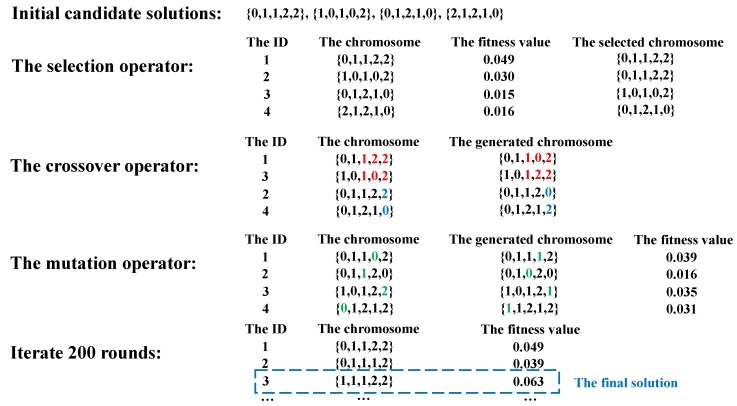
An AutoS example.

We use an example to explain the process of AutoS in detail. As shown in Figure 3, AutoS aims to find an optimal solution to assign each task to a computing node, with initial candidate solutions including 0,1,1,2,2, 1,0,1,0,2, 0,1,2,1,0 and 2,1,2,1,0. Their corresponding fitness values are 0.049, 0.030, 0.015 and 0.016, respectively. At the selection stage, AutoS selects chromosomes for further processing based on the probability that is proportional to the fitness values. Note that one solution may be selected for several times. At the crossover stage, the common section of two chromosomes may be exchanged randomly. For example, the chromosomes whose ids are 1 and 3 may exchange 1,2,3 and 1,0,2 to generate new chromosomes. At the mutation stage, the specific point of each chromosome will be replaced randomly by other values and then new chromosomes can be generated. For example, the 4th point of the 1st chromosome changes from 0 to 1. After these three stages, the fitness values are computed. Finally, after 200 iterations, 1,1,1,2,2 is returned as the final solution.    

**Algorithm 6:** AutoS **Input**: The number of initial scheduling solutions α, the elitism rate β, the mutation rate γ, the number of iterrations δ
 **Output**: A solution *X*1:set←∅, set_1←∅, set_2←∅, result←∅;2:set=generate_random(α);3:ETC=cal_ETC(set);4:**for**i=1 to δ **do**5: fit_value=cal_fitvalue(set,ETC);6: select_num←α*β;7: set_1←selection(set,select_num,fit_value);8: Select *Y* from set_1 with the highest fitness value;9: result←result∪Y;10: **for** j=1 to select_num **do**11:  x,y=random_select(set);12:  cross_point=random_point();13:  n_x,n_y=crossover(x,y,cross_point);14:  set_2←set_2∪(n_x,n_y);15: **end for**16: **for** j=1 to select_num **do**17:  mutate_point=random_point();18:  tmp=mutate(set_2,j,mutate_point,γ);19:  set_2=update(set_2,tmp,j);20: **end for**21: set←∅;22: set=set_1∪set_2;23:**end for**24:**return** The solution *X* with the highest fitness value in result.

Next, in the experimental section, we will evaluate the effectiveness and efficiency of AutoD, AutoE and AutoS, respectively.

## 5. Experiments

In this section, we evaluate the effectiveness and efficiency of our proposed algorithms on RHG 2018 dataset and BCTF 2019 dataset. AutoDES won the 7th and 5th place in RHG 2018 challenge and BCTF 2019 challenge, respectively.

### 5.1. Experimental Setup

#### 5.1.1. Evaluation Datasets

The following two datasets are used for evaluation.

- **RHG [52]** comes from the RHG 2018 challenge that was held in Wuhan, China in 2018. This dataset has 25 binary files, which can be divided into three classes that are *Stack Overflow*, *Format String Overflow* and *Heap Overflow*. AutoDES can exploit six binary files successfully.
- **BCTF-RHG [53]** comes from the BCTF-RHG 2019 challenge that was held in Beijing, China in 2019. This dataset has 20 binary files, which can be divided into six classes that are *Stack Overflow*, *Format String Overflow*, *Integer Overflow*, *Heap Overflow*, *Protocol Vulnerability* and *Logical Vulnerability*. AutoDES can exploit three binary files successfully.

The dataset statistics are shown in Table 2, where #NT and #NE represent the total number of binary files and the number of binary files that can be exploited by AutoDES, respectively.

#### 5.1.2. Comparison Methods

We use the following comparison methods for evaluation:

- In the stage of AutoD, **AFL [13]**, **AFLFast [29]**, **AFLGo [30]**, **Driller [11]** and **Anti-Driller** (described in Algorithm 2) are used.
- In the stage of AutoE, **Rex [7]**, **IPOV fuzzer**(described in Algorithm 3), **AutoJS** (described in Algorithm 4) and **AutoROP** (described in Algorithm 5) are used.
- In the stage of AutoS, **RR [38]** and **AutoS** (described in Algorithm 6) are used.

#### 5.1.3. Implementation Details

All algorithms are implemented in Python 2.7 and C++ and all of the experiments are conducted on Linux 16.04 with Core-i7 6700K CPU (4.00 GHz) and 64 GB main memory. We use Angr [54] to perform symbolic execution and static analysis on the programs. In the stage of AutoD, all the comparison methods are run for one hour, respectively.

### 5.2. Experimental Evaluation

#### 5.2.1. Analysis on AutoD

We evaluate the effectiveness of vulnerability discovery methods. As each method can produce multiple crashes for different binary files, we mainly focus on the first exploitable crash.

Table 3 shows the number of crashes discovered by different vulnerability discovery methods. AFL, AFLFast, AFLGo, Driller and Anti-Driller can produce the first exploitable crashes for 1, 2, 2, 2 and 2 binary files, respectively. Specifically, only the proposed Anti-Driller can produce the crashes for R4 and R8. The reason is that, to obtain the real vulnerabilities of R4 and R8, each method must pass a sanity check, which is very difficult to be tackled by fuzzing. Different from other methods, Anto-Driller uses CFG to skip this check and thus can obtain the real vulnerabilities. However, it still falls short in the effectiveness of the vulnerability discovery as other binary files have complex code structure, which may not be analyzed by Anti-Driller. We leave the further improvement on its performance to the future work.

We also observe that, for different binary files, due to different code structures, the time cost on the production of the first exploitable crash as well as the number of crashes discoverable by different vulnerability discovery methods are different.

#### 5.2.2. Analysis on AutoE

In this subsection, we evaluate the effectiveness of the vulnerability exploitation methods.

Table 4 shows the list of binary files that can be exploited by different vulnerability discovery methods. Rex, IPOV fuzzer, AutoJS and AutoROP can exploit 3, 4, 8 and 8 binary files, respectively. AutoJS and AutoROP outperform Rex and IPOV fuzzer, as Rex is designed for CGC and IPOV fuzzer is designed for binary files with simple code logic. However, the binary file B4 cannot be exploited by AutoROP as an ROP-chain doesn’t exist.

**Figure 4:**
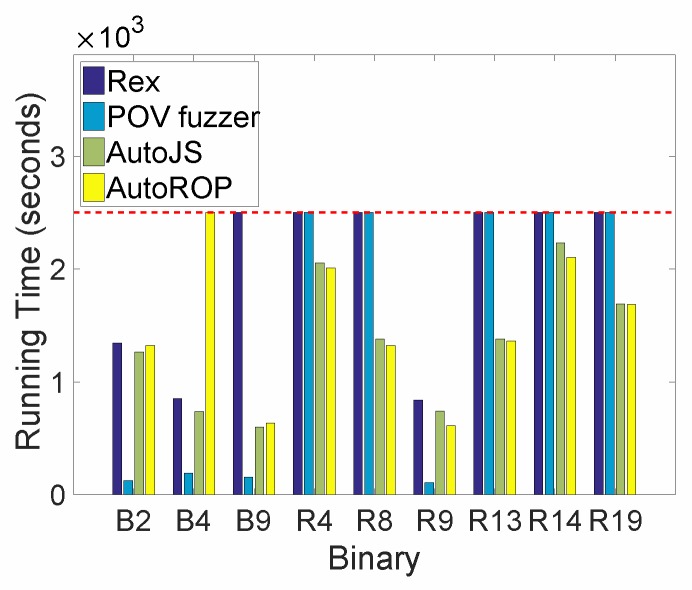
The running time of different vulnerability exploitation methods executed on different binary files. Each running time includes vulnerability discovery as well as exploitation time. Most importantly, those methods whose running time reaches $2.5\times 10^{3}$ s (see dashed red line) are deemed unfit with respect to the corresponding binary file.

Figure 4 shows the running time of different vulnerability exploitation methods executed on different binary files. For different binary files, the running time varies as different files have different code structure. Compared to other methods, the proposed IPOV fuzzer is the fastest one, although it cannot exploit all the binary files. AutoJS outperforms AutoROP in most cases.

In summary, we suggest that the IPOV fuzzer can be used as a prerequisite condition to determine whether a binary file can be exploited easily. If the IPOV fuzzer cannot exploit a binary file successfully, AutoJS and AutoROP can be called subsequently.

#### 5.2.3. Analysis on AutoS

In this section, we evaluate the effectiveness of the scheduling algorithm, AutoS. By fixing a specific ending running time, we compute the average process time for each task with different scheduling algorithms. In this experiment, we set the longest running time for each task to be six hours and every experiment is executed for 30 times.

**Figure 5:**
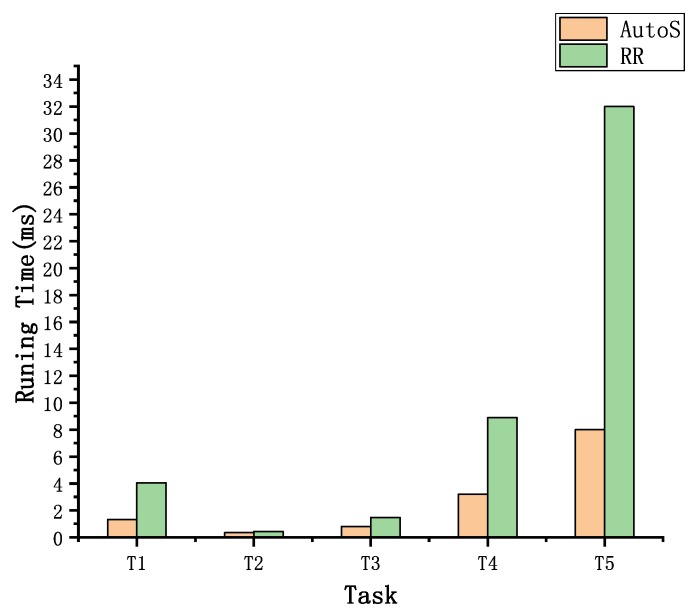
The average running time for each task with different scheduling algorithms executed by 30 times.

Figure 5 shows the average running time for each task with different scheduling algorithms. For each task, AutoS consumes less time and achieves better performances than RR. This demonstrates that AutoS is efficient in resource scheduling and can help to improve the efficiency of AutoDES.

### 5.3. A Case Study in IoT

In this section, a case study is given to explain the effectiveness of our framework. Our aim is to make the process of the vulnerability discovery and exploitation run automatically.

We first introduce the background of the case study. *MikroTik RouterOS* is an operating system for routers, which can help a commercial PC build the routing function. However, there exists a remote buffer overflow vulnerability (CVE-2018-7445) in this operating system that affects MikroTik RouterOS before versions 6.41.3/6.42rc27. As shown in Listing 4, the vulnerability is located on Lines 7–16. The first byte of the source buffer is read and used as the size for the copy operation. The function then copies that amount of bytes into the destination buffer. Once that is done, the next byte of the source buffer is read and used as the new size. This loop finishes when the size to copy is equal to zero. No validation is done to ensure that the data fits on the destination buffer, resulting in a stack overflow.

To restore the attack process and implement it automatically, we use the fuzzing tool *Mutiny Fuzzer* [55] designed by Cisco Talos to discover the vulnerability. As the operating system leverages the **Stack No-eXecute Protection** technique, we use AutoROP to exploit the vulnerability. The major party of the payload is listed in Listing 5.

**Figure 6:**
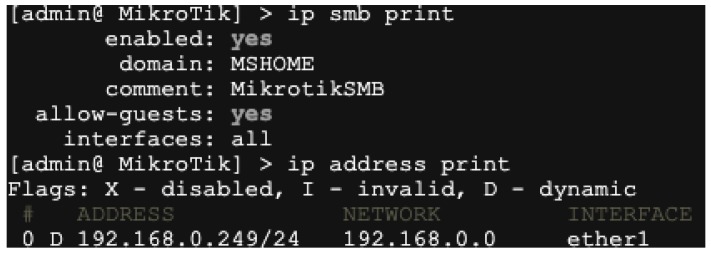
The successful exploitation of a remote Server Message Block (SMB) service of MikroTik RouterOS in Listing 4.

As shown in Figure 6, we successfully exploit the remote SMB service of MikroTik RouterOS in Listing 4, where we can print the IP address on the remote device. This case study demonstrates the effectiveness of our proposed framework in IoT.

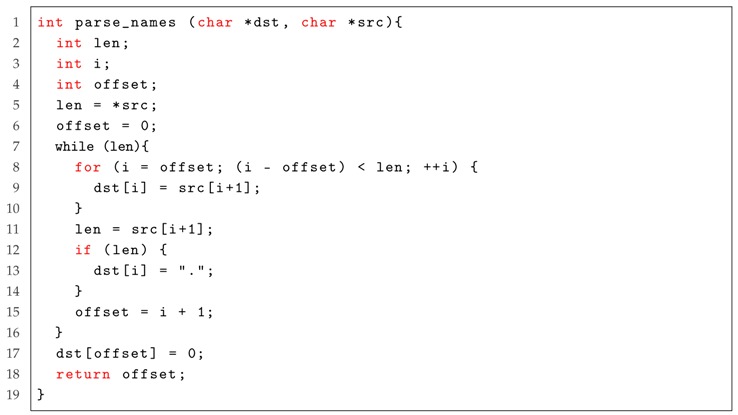
Listing 4: Case Study for the remote buffer overflow vulnerability in MikroTik RouterOS, where the highlighted part represents the location of the vulnerability.

In summary, the proposed framework is efficient and effective in vulnerability discovery and exploitation as well as resource scheduling.

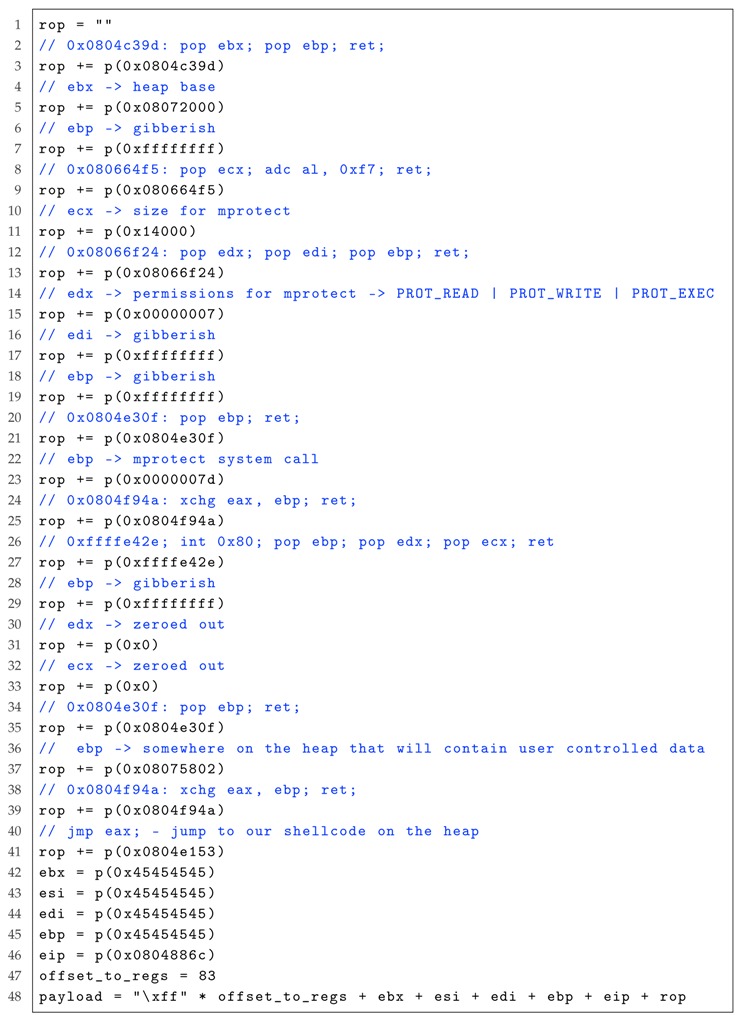
Listing 5: The major party of the payload for the case study listed in Listing 4.

## 6. Conclusions

In this paper, we propose an efficient and effective automatic vulnerability discovery and exploitation framework, AutoDES. In the stage of AutoD, we propose Anti-Driller to improve the effectiveness of vulnerability discovery. In the stage of AutoE, three attack methods IPOV fuzzer, AutoROP and AutoJS are proposed to improve the effectiveness of vulnerability exploitation. Moreover, we produce a genetic algorithm (GA)-based scheduling strategy (AutoS) that helps to assign the computing resources dynamically and efficiently, in turn drastically improving the efficiency of the overall framework. The comparative evaluation results demonstrate the effectiveness and efficiency of the proposed methods. As future work, we will focus on the following open problems. First, one potential direction is to further improve the effectiveness and efficiency of vulnerability discovery and exploitation. For example, we aim to exploit the binaries that have the *Heap Overflow* vulnerabilities. Second, it would be interesting to study how to use the proposed framework for binaries in the practical applications of IoT.

## Figures and Tables

**Table 1:** The comparison between the existing methods and our proposal AutoDES.

Methods		Similarity	Difference
AutoD	AFL [13] AFLFast [29] AFLGo [30] Steelix [31] T-Fuzz [32]	Both use fuzzing.	Anti-Driller uses dynamic symbolic execution.
	EXE [14] KLEE [15] SAGE [33] DART [34] CUTE [35] Smart-Fuzz [36]	They both use symbolic execution.	Anti-Driller also uses fuzzing.
	Driller [11]	They both use fuzzing and dynamic symbolic execution.	Anti-Driller also uses fuzzing.
AutoE	APEG [37]	They both generate exploits automatically.	APEG cannot support *Injecting a ShellCode*, *Return Oriented Programing* and *Jmp Esp* techniques.
	AEG [16]	They both generate exploits Automatically.	AEG needs a necessary manual preprocessing.
	Rex [7]	They both use symbolic execution.	Rex cannot be applied on the real-life applications.
AutoS	Round-Robin algorithm [38]	They both can allocate computing resources.	Round-Robin Scheduling algorithm falls short in efficiency.

**Table 2:** Graph statistics. The symbol “-” indicates that the dataset has no binary file with the specific vulnerability type. The content in the column “**Binary Files**" represents the names of binary files.

	RHG			BCTF-RHG		
	#NT	#NE	Binary Files	#NT	#NE	Binary Files
Stack Overflow	13	5	R4, R8, R9, R13, R14	7	2	B2, B9
Format String Overflow	2	1	R19	3	0	0
Integer Overflow	-	-	-	1	1	B4
Heap Overflow	10	0	0	7	0	0
Protocol Vulnerability	-	-	-	1	0	0
Logical Vulnerability	-	-	-	1	0	0

**Table 3:** The number of crashes found by different vulnerability discovery methods for each binary, where the parameters in the first line represent the binary ids. The number in the bracket indicates the time (minutes) that the first exploitable crash is produced.

Binary Id	B2	B4	B9	R4	R8	R9	R13	R14	R19
AFL	14	12	10 (9)	0	0	10	22	14	9
AFLFast	17	19	14	0	0	13 (9)	47 (21)	15	12
AFLGo	17 (17)	19	12	0	0	12	46	13	14 (27)
Driller	12	9 (10)	4	0	0	9	39	2 (34)	10
Anti-Driller	0	0	0	21 (32)	10 (22)	0	0	0	0

**Table 4:** The list of binary files that can be exploited by different vulnerability discovery methods. The symbols “√” and “×” represent whether a binary file can be exploited by a specific method or not, respectively.

Binary Id	B2	B4	B9	R4	R8	R9	R13	R14	R19
Rex	√	√	×	×	×	√	×	×	×
IPOV fuzzer	√	√	√	×	×	√	×	×	×
AutoJS	√	√	×	√	√	√	√	√	√
AutoROP	√	×	√	√	√	√	√	√	√
